# Insights Into *Histoplasma capsulatum* Behavior on Zinc Deprivation

**DOI:** 10.3389/fcimb.2020.573097

**Published:** 2020-11-30

**Authors:** Leandro do Prado Assunção, Dayane Moraes, Lucas Weba Soares, Mirelle Garcia Silva-Bailão, Janaina Gomes de Siqueira, Lilian Cristiane Baeza, Sônia Nair Báo, Célia Maria de Almeida Soares, Alexandre Melo Bailão

**Affiliations:** ^1^Molecular Biology and Biochemistry Laboratory, Institute of Biological Sciences II, Federal University of Goias (UFG), Goiania, Brazil; ^2^Laboratory of Experimental Microbiology, State University of Western Paraná (Unioeste), Cascavel, Brazil; ^3^Microscopy and Microanalysis Laboratory, Institute of Biological Sciences, Brasília University (UnB), Brasilia, Brazil

**Keywords:** fungal pathogenesis, Zn uptake, Zn and cell wall remodeling, proteomics, zinc homeostasis

## Abstract

*Histoplasma capsulatum* is a thermodimorphic fungus that causes histoplasmosis, a mycosis of global incidence. The disease is prevalent in temperate and tropical regions such as North America, South America, Europe, and Asia. It is known that during infection macrophages restrict Zn availability to *H. capsulatum* as a microbicidal mechanism. In this way the present work aimed to study the response of *H. capsulatum* to zinc deprivation. *In silico* analyses showed that *H. capsulatum* has eight genes related to zinc homeostasis ranging from transcription factors to CDF and ZIP family transporters. The transcriptional levels of *ZAP1*, *ZRT1*, and *ZRT2* were induced under zinc-limiting conditions. The decrease in Zn availability increases fungicidal macrophage activity. Proteomics analysis during zinc deprivation at 24 and 48 h showed 265 proteins differentially expressed at 24 h and 68 at 48 h. Proteins related to energy production pathways, oxidative stress, and cell wall remodeling were regulated. The data also suggested that low metal availability increases the chitin and glycan content in fungal cell wall that results in smoother cell surface. Metal restriction also induces oxidative stress triggered, at least in part, by reduction in pyridoxin synthesis.

## Introduction

*H. capsultatum* is a thermal dimorphic fungus that causes histoplasmosis, a mycosis with worldwide incidence and prevalence in temperate and tropical climates (Aide, 2009). This disease is widely distributed in Central and North America, mainly in the United States, Panama, and Honduras. In Latin America, a high incidence of the disease is seen in countries such as Brazil and Argentina. However, isolated cases have been reported in countries in Europe, Africa, and Asia (Bahr et al., 2015). Some molecular factors produced by *H. capsulatum* yeast cells enable them to parasitize phagocytic immune cells. Fungal yeasts are capable to infect and survive in phagocytic cells, including alveolar macrophages, polymorphonuclear leukocytes, and dendritic cells (De Sanchez and Carbonell, 1975; Schaffner et al., 1986; Gildea et al., 2001). These phagocytes serve as both the host cell and the vector by which infection dissemination is mediated. After phagocytosis, the fungus is exposed to stress conditions imposed by host defense mechanisms such as: deprivation of macro and micronutrients, production of reactive oxygen and nitrogen species, proteolytic enzymes, lipases, hypoxia and drastic changes in pHs. Among those, zinc deprivation has been identified as a microbicidal strategy used by host cells to control *H. capsulatum* infection (Garfoot and Rappleye, 2016; Shen and Rappleye, 2017).

Zinc is an important chemical element in all life kingdoms. This ion has different chemical characteristics from other metals because of its high ionization potential that favors the formation of covalent bonds (Alberts, 1998; Permyakov, 2009). This property allows the zinc to binds to two main protein classes: enzymes, in which constitute 3/5 of the zinc metalloproteins and transcription factors (Andreini et al., 2006; Andreini et al., 2009). Zinc-binding enzymes are distributed in all cellular components, with an emphasis on cytoplasm and mitochondria, where most of the metabolic processes occur (Tristão et al., 2014). In addition, zinc, in its free form within the cell, acts as an activator of cell signaling, regulating transporters that have the ability to capture zinc during deprivation or stress conditions (Auld, 2001; Permyakov, 2009). Thus, it is clear that small changes in zinc concentrations may alter cellular metabolism (Taylor et al., 2012). In *H. capsulatum*, the metabolic response during zinc deprivation is not well known. However, it is known that zinc is essential for the survival of this fungus during the infection process. Studies by Vignesh et al. (2013) showed that *H. capsulatum* undergoes zinc deprivation when inside of GM-CSF activated macrophages. One of the mechanisms of zinc deprivation controlled by GM-CSF exposed macrophages is the production of metallothioneins specific to this metal, which ends up reducing the amount of free zinc in the fungus (Vignesh et al., 2013). As a result, it is suggested that *H. capsulatum* developed zinc capture and storage strategies to increase the chances of survival in phagocytic cells (Shen and Rappleye, 2017).

The fungus defense against zinc deprivation is the activation of a very specific transcription factor, known as Zap1, which regulates zinc homeostasis. Zap1 and its orthologs have already been characterized in several fungal species such as *Saccharomycces cerevisiae* (Frey et al., 2011)*, Cryptococcus gattii* (Schneider et al., 2015)*, Aspergillus fumigatus* (known as ZafA) (Vicentefranqueira et al., 2018), and *Candida Albicans* (initially named Crs1) (Kim et al., 2008). This transcription factor increases the expression of the ZIP (Zrt/Irt type Proteins) and CDF (Cation Diffusion Facilitators) family of transporters capable of transporting zinc towards the cytoplasm or organelles, respectively. In some organisms, such as in *S. cerevisiae*, Zap1 is capable of auto regulation, inducing itself and also a range of proteins involved on the adaptation to different levels of zinc (Schneider et al., 2012; Wilson et al., 2012; Amich and Calera, 2014). In *H. capsulatum*, a member of the ZIP family (Zrt2) has been characterized. *HcZrt2* behaves as a high affinity transporter, being necessary for growth in zinc limiting condition as well as playing an important role during infection *in vivo* (Dade et al., 2016).

In some fungal species, there are also non-specific metal carriers or even different transcription factors. Fet4 and Pho84 for example, have binding sites for iron, copper, zinc, manganese, and magnesium. These transporters assist Zrts in regulating zinc homeostasis, where they also capture zinc from the extracellular medium to the intracellular medium. Loz1 is the only transcription factor that seems to behave similarly to Zap1 in fungi, capable of regulating homeostasis in zinc replete conditions in *Schizosaccharomyces pombe* (Corkins et al., 2013). In addition to plasma membrane transporters or transcription factors, there are also intracellular carriers, such as Zrc1, Cot1, and Zrt3 in *S. cerevisae*. The first two store zinc into the vacuole when the fungi is in conditions of high zinc availability or when preparing for “zinc shock”, a term applied when the environment rapidly shifts from deplete to replete zinc condition in a short span. Zrt3 meanwhile, has an inverse function, in which it is able to export zinc stored in the vacuole to the cytoplasm when the environment has low availability of this metal (Eide, 2006; Wilson et al., 2012).

Unlike *S. cerevisiae* and *C. neoformans*, some fungi have alternative mechanisms for zinc homeostasis, being regulated not only by zinc, but by pH as well. Examples of this can be seen in *Aspergillus fumigatus* and *Candida albicans* (Vicentefranqueira et al., 2005; Wilson et al., 2012). In *A. fumigatus*, there are 18 genes related to zinc transport within the cell, of which two are transcription factors, ZafA (homologous to Zap1) and PacC, that, while not induced by zinc but rather pH, PacC influences expression of some of the remaining zinc transporters from the ZIP and CDF family (Moreno et al., 2007; Amich and Calera, 2014; Bertuzzi et al., 2014). *C. albicans*, has an extracellular zinc capture mechanism mediated by Pra (pH-regulated antigen 1) that has a high affinity for zinc and plays a role in alkaline conditions. It is known that this molecule is secreted in the extracellular medium to capture free zinc in the host cytoplasm or from storage proteins. Subsequently, the Pra–Zn complex makes the captured metal available to the cytoplasm through Zrt1 (Wilson et al., 2012). Pra1 was later found in *A. fumigatus* (named Aspf2) and *Blastomyces dermatitidis*, being also associated with a specific ZIP transporter, ZrfC and Zrt1, respectively.

Besides the control of zinc concentrations inside the cell, Zap1 also regulates multiple proteins involved in maintaining metabolic homeostasis such as enzymes related to aerobic metabolism, fermentation, and the biosynthesis of secondary compounds (Wilson and Bird, 2016). In addition to those, over 500 proteins linked to this metal have already been identified, and approximately 70% are enzymes (Permyakov, 2009). In *H. capsulatum*, current understanding of how those processes operate or what proteins are involved in the maintenance of optimal zinc concentrations is currently lacking. In the present work, we sought to elucidate *H. capsulatum’s* general behavior during zinc starvation through genomic analysis and proteomic approaches.

## Materials and Methods

### *In Silico* Analysis

Protein sequences of genes related to zinc homeostasis were obtained in Gene bank (https://www.ncbi.nlm.nih.gov/protein). The *in silico* homology analyses were performed using the tool BLASTp (Basic Local Alignment Search Tool) (https://blast.ncbi.nlm.nih.gov/Blast.cgi). Sequences of zinc homeostasis proteins of *A. fumigatus*, *C. neoformans, P. brasiliensis*, and *S. cerevisiae* were used as templates. Additionally, STRING (https://string-db.org/) was used in analyses of protein interactions in *H. capsulatum* Nam1 and the BLASTp tool was used to find orthologs in G186AR strain.

### *H. capsulatum* Growth Conditions

*H. capsulatum* yeasts (G186AR, ATCC26029) were maintained in a solid chemically defined medium McVeigh/Morton (MMcM) (Restrepo and Jimenez, 1980), supplemented with 20 μM ZnSO_4_, 200 μM FeSO_4_, and 30 μM CuSO_4_ at 37°C under an atmosphere of 5% CO_2_ for seven days. For zinc-limiting assays the yeast cells (10^8^ cells/ml) were incubated in liquid MMcM with no zinc and supplemented with 100 μM of zinc chelator DTPA (diethylenetriaminepentaacetic acid, Sigma Aldrich), 200 μM FeSO_4_, and 30 μM CuSO_4_ to avoid unspecific metal-chelation promoted by DTPA, under agitation for 24 or 48 h. Cells incubated in MMcM with 20 μM of ZnSO_4_ were used as controls. For analyses of pyridoxine on *H. capsulatum* growth, yeast cells were incubated under Zn deprivation and different concentrations of pyridoxine (0, 1, and 6 μM). The growth of *H. capsulatum* in liquid culture was quantified by measurement of culture turbidity using optical density (OD 595 nm). The cellular viability of *H. capsulatum* yeast cells was determined by the trypan blue method (Trypan Blue, Sigma Aldrich) using a Neubauer chamber at optical microscopy. The growth analyses were performed in triplicate.

### RNA Extraction and qRT-PCR

The expression analysis of Zn homeostasis genes was performed by quantitative real time reverse transcription polymerase chain reaction (qRT-PCR) using cells grown during 3 and 24 h in control (MMcM supplemented with 20 μM of ZnSO_4_) and Zn-limiting conditions. The cells were harvested and subjected to total RNA extraction by mechanical cell rupture using a BeadBeater (Mini-BeadBeather, Biospec Products Inc., Bartlesville, OK, USA), 0.5 μm diameter glass beads and TRIzol (TRI Reagent^®^, Sigma-Aldrich, St. Luois, MO, USA), according to the manufacturer’s protocol. The RNAs were used to synthesize single stranded cDNAs (DNA complementary) using the High Capacity RNA-to-cDNA kit (Applied Biosystems, Foster City, CA) and oligo (dT) primer. The qRT-PCR was performed using SYBER green PCR master mix (Applied Biosystems, Foster City, CA) on the Applied Biosystems Step One Plus Real-Time PCR System (Applied Biosystems Inc.) with 10 pmol of each specific primer and 40 ng of template cDNA in a final volume of 25 μl. Melting curve analysis was performed to confirm a single PCR product. Standard curves were generated using 1:5 serial dilutions by pooling cDNAs of all conditions. The qRT-PCR assays were performed in triplicates. The levels of relative expression of the transcripts were calculated using the standard curve method for relative quantification (Bookout et al., 2006). The oligonucleotides used in the real time PCR analysis were constructed based on the structural genome of *H. capsulatum* (https://www.ncbi.nlm.nih.gov/protein, **Supplementary Table 1**).

### Proteomic Analysis

Protein extracts of *H. capsulatum* were obtained from yeast cells lysed using a BeadBeater (Mini-BeadBeather, Biospec Products Inc., Bartlesville, OK, USA), 0.5 μm diameter glass beads and ammonium bicarbonate buffer (pH 8.5). The cells were disrupted six times for 30 s at maximum speed with 1 min intervals on ice. Then, the lysate was centrifuged five times at 10,000 × *g* for 10 min at 4°C to remove cell debris. The protein extracts were quantified using the Bradford method (BRADFORD, 1976) with bovine serum albumin solution as standard. The enzymatic digestion was performed based on Murad et al. (2011), using 150 μg of total protein extract in ammonium bicarbonate buffer (50 mM) and RapiGEST™ SF (Waters, Milford, MA, USA) for 15 min at 80°C. Then, dithiothreitol (DTT, GE Healthcare, Little Chalfont, UK, 100 mM) was added and samples incubated for 30 min at 60°C followed by addition of iodoacetamine (300 mM, GE Healthcare, Piscataway, NJ, USA) and incubation at room temperature in a dark room. The digestion was done by the addition of 30 μl of a 0.05 μg/μl trypsin solution (Promega, Madison, WI, USA) and incubation at 37°C for 16 h. Subsequently, 10 μl of 5% (v/v) triofluoracetic acid (TFA) was added, followed by incubation at 37°C for 90 min. The samples were centrifuged, and supernatant was lyophilized in the speed vacuum (Eppendorf, Hamburg, Germany). The tryptic peptides were separated by Ultra High Performance Liquid Chromatography according to Baeza et al. (2017), using the ACQUITY UPLC^®^ M-Class system (Waters Corporation, Manchester, UK) coupled to Synapt G1 HDMS™ mass spectrometer (Waters Micromass, Manchester, UK). Rabbit phosphorylase B (MassPREP™ Digestion Standard) was used as internal standard and [GLU1]-Fibrinopeptide B (GFB) for calibration during the sample analysis. Mass spectra were processed using the ProteinLynx Global Server software version 3.0.2 (Waters, Manchester, UK) loaded with a specific database for *H. capsulatum* (Geromanos et al., 2009). Proteomic analyses were performed in triplicates for each sample. Identified proteins with at least 1.2 fold change difference between conditions were considered as regulated. Subsequently, online tools such as FungiDB (https://fungidb.org/fungidb/) and KEGG (https://www.genome.jp/kegg/pathway.html) were used to functionally categorize the regulated proteins.

### Carbohydrate and Glucose Dosage

Total carbohydrate content from yeast cells grown in control and DTPA conditions was measured using the modified phenol-sulfuric acid reaction (Dubois et al., 1956). A harvest pellet from an aliquot of 1 ml culture sample was mixed with 800 µl of concentrated sulfuric acid (98%) and 50 µl of 80% phenol aqueous solution in a microtube. The samples were read at 490 nm on a 96 well microplate spectrophotometer. A standard curve was constructed using a serial dilution of a glucose solution (5 mg/ml) in water. The values were normalized, and the concentration was expressed in micrograms of carbohydrate per gram of cell dry weight. The assays were conducted in triplicate. The glucose concentration in culture supernatants was determined using Bioclin Glucose Monoreagent kit (cat. no. K082, QUIBASA QUIMICA BÁSICA Ltda, Belo Horizonte, Brazil). *H. capsulatum* yeast cells (10^8^cells/mL) were incubated under control and DTPA conditions, during 0, 24, 48, and 72 h. Then, the cells were centrifuged at 1,371 × *g*, and the supernatant was used for glucose measurement according to the manufacturer’s instructions. A standard curve was constructed using MMcM medium with crescent glucose concentrations (0, 0.125, 0.250, 0.50, 0.75, and 1%). The dosages were performed in triplicate.

### Quantification of Chitin, Glucan and ROS Content

*H. capsulatum* cells grown in control and Zn-limiting conditions were collected by centrifugation at 1,372 × *g* and washed with PBS. For chitin dosage the cells were treated for 30 min with calcofluor white (100 μg/ml). For glucan dosage the cells were treated for 5 min with aniline blue. After treatment, the cells were washed twice with PBS and analyzed under fluorescence microscope (Zeiss Axiocam MRc-Scope A1). Intracellular peroxide hydrogen (H_2_O_2_) was measured in *H. capsulatum* yeasts incubated under conditions of zinc and pyridoxine deprivation for 24 h. The fluorescence was obtained using 2′7′-dichlorofluorescein diacetate (DCFH-DA, 25 nM) for 25 min in the dark. The fluorescence intensity of yeast cells was measured using the AxioVision software (Carl Zeiss AG, Germany). The minimum of 30 cells for each microscope slide was used to calculate fluorescence intensity (in pixels). The assays were performed in triplicate.

### Scanning Electron Microscopy

The *H. caspulatum* yeast cells were fixed with a solution containing 2% (v/v) glutaraldehyde and 2% (w/v) paraformaldehyde in 0.05 M sodium cacodylate buffer pH 7.4 overnight at room temperature. After, the cells were washed in sodium cacodylate buffer pH 7.2 and incubated for 1 h in 2% (w/v) osmium tetroxide (OsO_4_) and 0.8% (w/v) potassium ferricyanide in 0.05 M sodium cacodylate buffer pH 7.4. Subsequently, the cells were washed to remove excess osmium tetroxide and dehydrated in ascending series of acetone solutions (v/v) ranging from 30, 50, 70, 90, to 100%. The samples were dried by the critical point Balzers CPD 30 and placed in stubs to be coated with gold in a Sputter Coater Balzers SCD 050 and then examined in a scanning electron microscope (7001F, JEOL, Tokyo, Japan).

### Alcohol Dehydrogenase Activity

Fresh cell extracts from *H. capsulatum* yeasts incubated in control and Zn-limiting conditions were used to measure alcohol dehydrogenase (ADH) activity according to the NADH consumption at 350 nm. Briefly, 5 μg of protein extract was added to 180 ul of reaction mixture (100 mM MES buffer pH 6.1, 1 mM DTT, 5 mM MgCl_2_, 0.2 mM NADH, and 20 mM acetaldehyde). The reactions were measured every 10 s over 10 min in a microplate reader. The ADH measurement assays were performed in triplicate.

### Macrophage Infection Assays

Macrophages J774 1.6 were supplied by the Rio de Janeiro Cell Bank (BCRJ) of the Federal University of Rio de Janeiro (UFRJ). Macrophages were maintained in RPMI 1640 medium (Biowhittaker, Walkersville, Md.) supplemented with 10% (v/v) fetal bovine serum (FBS) (Citrocell/Embriolife, Campinas, SP, Brazil), 1% (v/v) essential amino acid, and 50 ug/ml of gentamicin solution (Sigma Biochemical) at 36°C and 5% CO_2_ until completely confluent. For fungal burden analysis, macrophages (3.75 × 10^6^) cells were plated in 24-well plates (Greinner Bio-One, USA) with RPMI medium containing IFN-*γ* (1 U/ml) (Sigma Aldrich) for 24 h followed by incubation with RPMI medium containing DTPA or 20 μM of ZnSO_4_ for 1 h. The cells were then washed twice with PBS. A control sample was incubated with the maintenance medium. After 1.8 × 10^7^ *H. capsulatum* yeasts (MOI 1:5) were added to each well, and the plates were incubated for 24 h at 36°C and 5% CO_2_. Each well was then washed with PBS, the macrophages were lysed with sterile cool water and the lysates plated on solid MMcM plates. The experiments were performed in triplicate.

### Statistical Analysis

The data were analyzed using electronic spreadsheets and the statistical software R. In the present work, the t Student statistical test was used to analyze gene expression, proteomics, and microscopy data. Meanwhile, CFU’s statistical tests were performed through analysis of variance (ANOVA) followed by Tukey test. In addition, in order to verify the quality of the proteomic data, multivariate principal component analysis was applied. Finally, factor analysis was performed with proteomic data to identify proteins regulated by treatment and time.

## Results

### Growth and Fungal Viability in Zinc Deprivation

**Figure 1:**
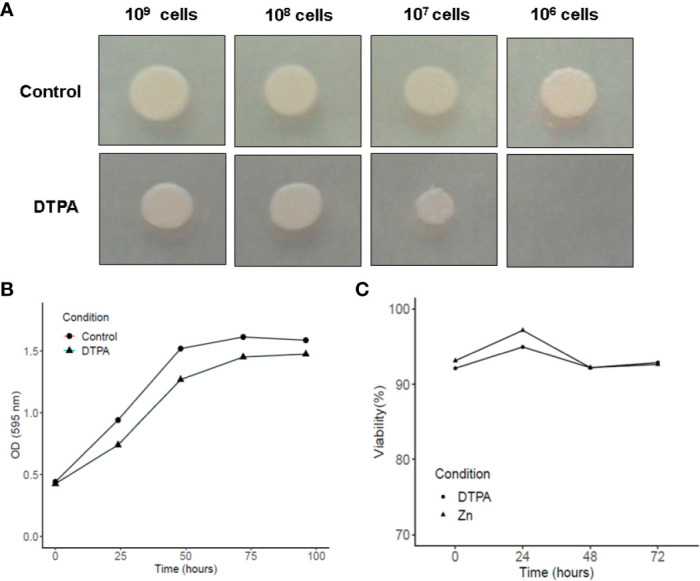
Influence of Zn availability on *H. capsulatum* growth and viability. **(A)** Growth in solid MMcM medium during 5 days with 20 μM of ZnSO_4_ or 100 μM DPTA. **(B)** Growth of *H. capsulatum* in liquid medium was measured by optical density at 595 nm. **(C)** Viability of *H. capsulatum* in liquid medium was assessed by trypan blue dye. Growth and viability experiments were carried out in biological triplicates.

Since zinc limitation is one of the GM-CSF-activated macrophage strategies to control *H. capsulatum* infection (Vignesh et al., 2013), fungal growth was assessed in a zinc-limited environment. Spot dilution growth analysis infers a slight reduction in growth when the medium is supplemented with DTPA (**Figure 1A**). The treatment with DTPA did not affect the fungal growth in liquid medium. The growth differences between solid and liquid media may be explained by the fact that in solid medium the cell-nutrient contact is dependent on diffusion, which is less efficient then in liquid cultures. Also, the yeast cell viability is not affected by DTPA treatment (**Figure 1C**). Based on these findings, it may be concluded *H. capsulatum* is able to grow in a zinc poor environment and thus presents molecular machinery to deal with it, at least *in vitro*. Our data is consistent with previous findings that have shown DTPA treatment decreases fungal growth as assessed by tritiated leucine uptake assays (Winters et al., 2010).

### Data Mining of Zinc Homeostasis Related Genes in *H. capsulatum* Genome

**Figure 2:**
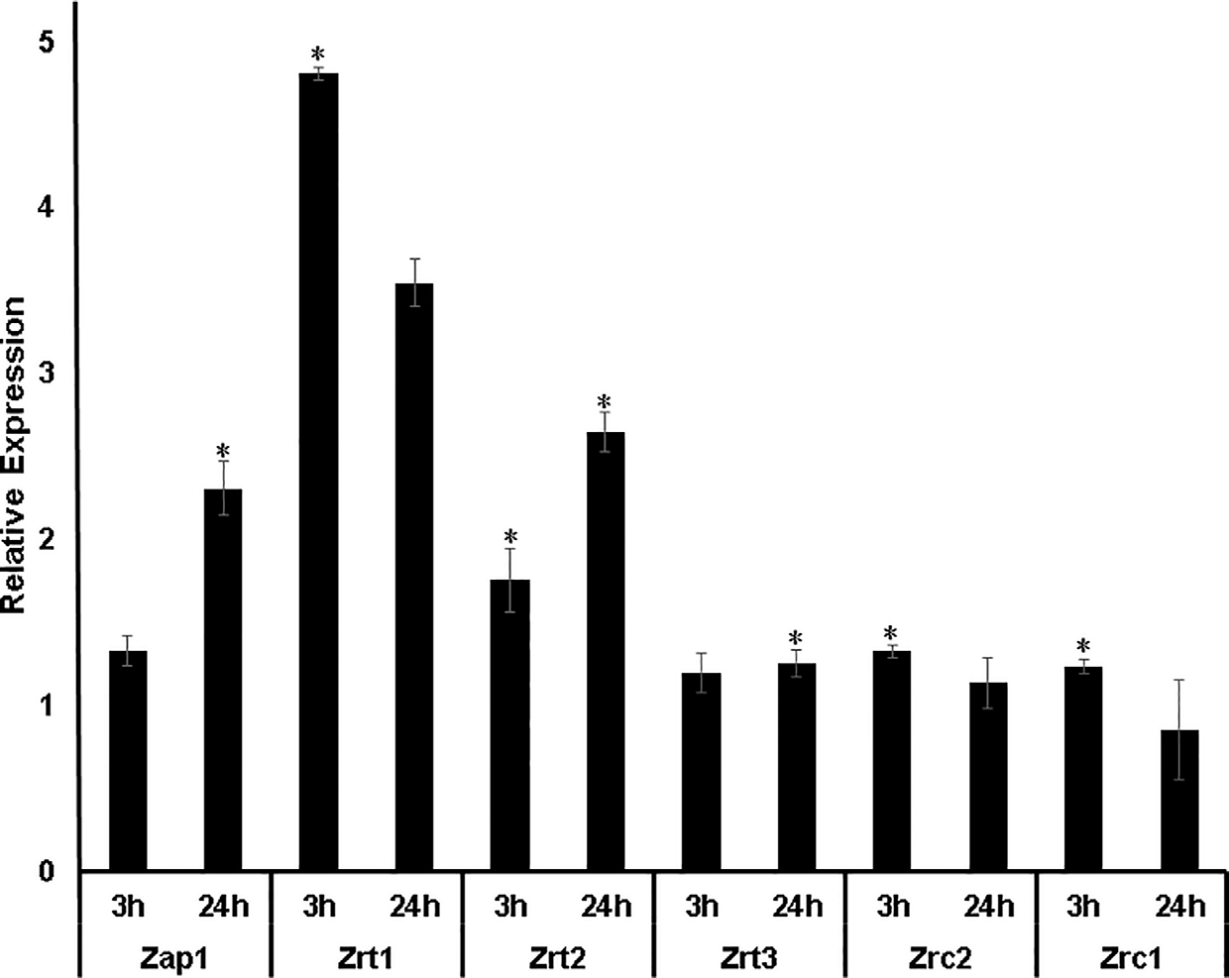
Transcript levels of Zn homeostasis genes during Zn deprivation. ZAP1: transcription factor that regulates the expression of zinc transporters. ZRT11, ZRT2, and ZRT3: Zinc carriers of the ZIP family. ZRC1 and ZRC2: Zinc transporters of the CDF family. The DTPA transcript levels were normalized against the transcript levels of control condition. Transcriptional levels of Zn-related genes were measured in biological triplicates. *:*p*-value ≤ 0.05.

The genome of *H. capsulatum* G186 was screened for genes involved in the coordination of zinc content inside the cell and, with it, maintaining safe zinc levels (zinc homeostasis). The search was performed using genes already characterized in other fungi. The approach led to the identification of eight genes putatively related to zinc homeostasis (**Table 1**). Two transcription factors were found, Zap1 with an identity average of 56.8% and PacC with an identity average 62.8% when compared to regulators of other fungi. These two transcription factors have joint action in regulating zinc uptake transporters in different pH environments. In addition, six zinc transporters were identified in the *H. capsulatum* genome, three belonging to the ZIP family. The first one was initially named *Zrt2* (Dade et al., 2016). However, experimental analysis showed that it behaves as *ZRT1* found in *S. cerevisiae*, being highly induced during zinc deprivation (**Figure 2**); hence, we saw it fit to name it accordingly. The second ZIP transporter holds a high identity to *A. fumigatus ZrfC* (60%). *ZrfC* is a transporter expressed at low zinc availability and high pH environments and it is tightly associated with a zinc extracellular captor (*Aspf2* in *A. fumigatus* and *PRA1* in *B. dermatitidis)* (Citiulo et al., 2012). Curiously, *PRA1*/Aspf2 was not found in the *H. capsulatum* genome. Furthermore, *HcZrfC* ortholog showed no apparent pH dependent regulation (data not shown). Given that, this transporter was named Zrt2, as so far it was shown to behave in a similar fashion to *ZrfA*, the low affinity zinc transporter in *A. fumigatus* (Vicentefranqueira et al., 2005). The remaining ZIP transporter showed significant identity (ranging from 41 to 74%) to *ZRT3*, the zinc exporter found in the vacuole (Wilson et al., 2012). The CDF family in *H. capsulatum* seems to be composed of *ZRC1*, the known vacuole exporter, *ZRC2*, a *ZrcA* ortholog in *A. fumigatus* (52% identity) and *ZITB*, a CDF found in *E. coli* (Grass et al., 2001), with its biological function not yet characterized in fungi.

**Table 1:** Identity of zinc homeostasis related genes of *H. capsulatum* G186 when compared to functionally characterized genes.

Accession number	Gene	*P. lutzii (Pb01)*	*P. brasiliensis (Pb03)*	*B. dermatitidis (ER-3)*	*C. immitis (RS)*	*A. fumigatus (Af293)*	*S. cerevisiae (S288C)*
HCBG_07321	*ZRT1*	62% (XP_002789372.1)	63% (EEH18665.1)	68% (EEQ92299.1)	63% (XP_001240918.1)	66% (XP_749518.1)	43% (NP_013231.1)
HCBG_08608	*ZRT2*	68% (XP_002794874.1)	68% (EEH17011.2)	78% (EEQ91531.1)	55% (XP_001241167.2)	51% (XP_751869.1)	30% (NP_013231.1)
HCBG_04549	*ZRT3*	65% (XP_015701767.1)	65% (EEH22486.1)	–	56% (XP_001247051.1)	49% (XP_755208.1)	41% (NP_012746.1)
HCBG_02465	*ZRC2*	70% (XP_015700796.1)	70% (EEH17306.2)	66% (EEQ87926.2)	–	54% (XP_748854.2)	30% (NP_014961.3)
HCBG_00193	*ZRC1*	69% (XP_015703463.1)	68% (EEH19201.2)	–	63% (XP_001240418.1)	61% (XP_755789.1)	48% (NP_013970.1)
HCBG_07376	*PACC*	61% (XP_015700598.1)	–	77% (EEQ84340.1)	61% (XP_001244284.1)	59% (XP_754424.1)	55% (NP_011836.1)
HCBG_03275	*ZAP1*	62% (XP_015701851.1)	63% (EEH21074.2)	68% (EEQ90390.2)	44% (XP_001239281.2)	65% (XP_752374.1)	45% (NP_012479.1)
HCBG_07983	*ZITB*	42% (XP_015701946.1)	45% (EEH17028.2)	53% (EEQ87344.2)	57% (XP_001249104.2)	64% (XP_751291.1)	41% (NP_010491.4)

-ortholog not found in H. capsulatum genome.

In addition, the STRING tool was used in order to analyze the interaction of Zap1 with other proteins related to zinc homeostasis. With it, a network of interactions of the regulator with proteins related to Zn homeostasis was found (**Supplementary Figure 1**). The network shows interactions between Zap1 and Zrt1, as well as Zrt3. Furthermore, the network also showed interaction of Zap1 with transporters probably present in the membrane of organelles such as Zrc1 and Zrc2 that, under conditions of zinc availability, capture and store this metal in vacuoles. Interestingly, ZitB, a zinc carrier of the CDF family, was also found in the interaction network. By using the STRING approach, interactions between carriers Zrt1 and Zrt3 (ZIP family) with carriers of the CDF family (Zrc1 and Zrc2) were also identified. Similar analysis with PacC revealed no interactions with Zn-related genes.

### Expression of Zinc Homeostasis Genes During Zinc Deprivation

The influence of Zn availability on transcriptional levels of *H. capsulatum* genes putatively related to Zn homeostasis was analyzed by qRT-PCR (**Figure 2**). The transcription factor *ZAP1*, genes from the ZIP family (*ZRT1, ZRT2, ZRT3*), and two genes from the CDF family (*ZRC2 and ZRC1*) were analyzed. qPCR assays showed that *ZAP1* transcripts increased after 24 h of Zn deprivation. The levels of *ZRT1* and *ZRT2* transcription were induced under Zn limiting conditions at both time points. *ZRT1* stands out as it was the transporter that presented the highest levels of expression among all transporter-genes analyzed. In comparison with *ZRT2*, *H. capsulatum ZRT1* showed a threefold increase in expression after 3 h exposure to a low zinc environment. In addition, *ZRT3* showed a significant difference only in zinc deprivation after 24 h when compared to control (**Figure 2**). These changes in gene expression are due to the Zn-deprivation promoted by DTPA since when Zn is added back to DTPA treated cells the *ZRT1* expression level decreases to control the cells (**Figure S2**). Also, the changes promoted by DTPA are not due to chelation of other metals since DTPA does not regulate the expression levels of a high affinity copper transporter (*CTR3*, **Figure S2**). The fact that Zap1 mRNA levels increase at 24 h but its putative regulated transporter is induced at 3 h of Zn deprivation suggests that *H. capsulatum* already has baseline levels of *ZAP1* that acts quickly by increasing *ZRT1* expression. Although surprising, the non-concurrent ZAP1 induction compared to its regulated transporters is not uncommon. Such claims are validated by similar findings in different fungi such as *C. dubliniensis* (Bottcher et al., 2015). Therefore, these results suggest that carriers of the *H. capsulatum* ZIP family are regulated by the amount of zinc available in the cell. Regarding the genes of the CDF family, Zn depletion promotes slight changes in transcript levels of Zrc1 and Zrc2 transporters. Thus, the role of these genes on zinc homeostasis in *Histoplasma* must be approached by mutant based assays.

### Influence of Zinc in *H. capsulatum* Survival in Macrophages

**Figure 3:**
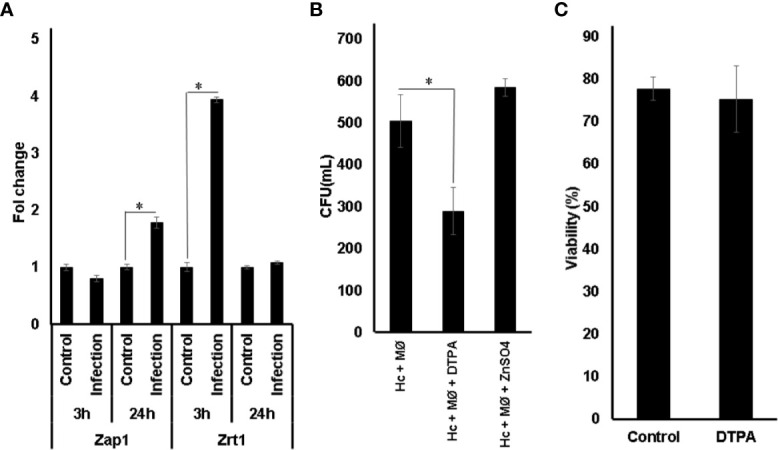
Influence of Zn availability in *H. capsulatum* survival in macrophages. **(A)** Relative expression of Zap1 and Zrt1 in *H. capsulatum* during infection overtime. **(B)** Fungal burden of macrophages treated with (Hc + MØ), ZnSO4 (Hc + MØ + ZnSO4), and DTPA (Hc + MØ + DTPA) conditions. Control: *H. capsulatum* incubated in macrophage medium; Infection: macrophages infected with *H. capsulatum* yeasts. **(C)** Viability of macrophages in RPMI medium was measured by trypan blue dye. Statistical analysis was performed using t-test, one-way ANOVA, and the Tukey multiple comparison test with *p ≤ 0.05. All experiments were carried out in triplicates.

Transcript levels of *ZAP1* and *ZRT1* were measured during macrophage infection (**Figure 3A**). Since IFN-*γ* plays an essential role in fungicidal activity of macrophage (Allendoerfer and Deepe, 1997; Horwath et al., 2015), cells were pre-treated before infection. The data obtained showed that both Zap1 and Zrt1 were induced in fungal cells growing inside macrophages. These findings reinforce previous studies showing that *H. capsulatum* undergoes a Zn limiting environment inside GM-CSF exposed phagocytes (Vignesh et al., 2013). In order to confirm the influence of Zn availability on fungus survival during infection, colony-forming unit analysis was performed (**Figure 3B**). Pre-treatment of macrophages with DTPA reduces fungal survival. This fact is not an effect of DTPA on macrophages, since the viability of the cells is not affected by DTPA (**Figure 3C**). These data together with the increased expression of *ZAP1* and *ZRT1* suggest that *H. capsulatum* faces a low zinc environment as a microbicidal strategy of macrophages and counteracts it, activating high affinity Zn-uptake mechanism.

### Proteomic Analysis of *H. capsulatum* During Zinc Deprivation

**Figure 4:**
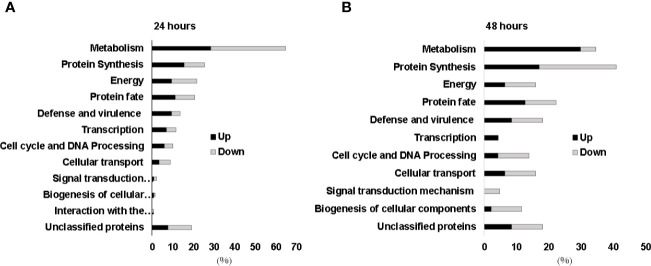
Functional categorization of proteins identified during zinc deprivation at 24 h **(A)** and 48 h **(B)**. Up, upregulated proteins during zinc deprivation. Down, downregulated proteins during zinc deprivation.

Since *H. capsulatum* is able to grow inside the intraphagosomal Zn-poor environment, the global response of the fungus to metal scarcity was accessed through proteomics. The approach identified 333 proteins regulated during zinc deprivation, 265 and 68 after 24 h and 48 h of treatment, respectively. According to statistical analysis, 119 proteins were induced at 24 h, and 146 were suppressed (**Supplementary Tables 2** and **3**), while at 48 h 47 proteins were induced and 21 repressed during zinc deprivation (transporter-genes). After 24 h, metabolic pathways related to fatty acid oxidation, energy production, oxidative phosphorylation, amino acid catabolism, biosynthesis of secondary metabolites (pyridoxine), and response to oxidative stress were induced, while glucose oxidation pathways, glyoxylate cycle, and fermentation were repressed (**Figure 4A**). Pathways with the greatest number of induced regulated proteins include fatty acid oxidation, energy production, oxidative phosphorylation, and cell wall carbohydrate biosynthesis (Chitin and Glucan), all after 48 h of Zn deprivation. Meanwhile, glycolysis and fermentation remained suppressed (**Figure 4B**).

In order to filter proteins regulated by the absence of zinc along time, a factor analysis was performed. With it, 22 proteins appeared under regular (control) and zinc deprivation conditions in both times (24 and 48 h) and therefore were included in the analysis (**Supplementary Figure 4**). The approach identified three proteins (Prefoldin subunit 4, Tyrosine Kinase, and Glyoxylate reductase) differentially regulated by zinc deprivation and also by time (**Table 2**). It is worth mentioning that glyoxylate reductase is increased at 24 h of zinc deprivation and is decreased at 48 h. Thus, it is likely that the enzyme produces the necessary amounts of precursors for the biosynthesis of other metabolites in zinc deprivation in the first 24 h, and therefore, this enzyme is important for early fungal adapting to Zn deprivation.

**Table 2:** Core proteins identified regulated by zinc and time.

Accession number	Protein	Control 24–48 h	DTPA 24–48h
HCBG_03816	Prefoldin subunit 4	0.34	0.05
HCBG_05072	Tyrosine-protein kinase	0.82	0.0000006
HCBG_03370	Glyoxylate reductase	0.83	0.0000019

Control 24**–**48 h, p values of the factor analysis for control conditions regulated over time. DTPA 24**–**48 h, p values of the factor analysis for DTPA condition regulated over time.

**Figure 5:**
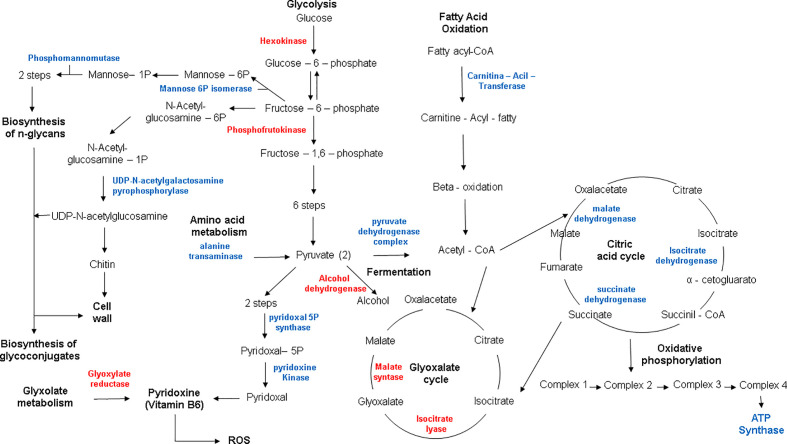
Metabolic profile of *H. capsulatum* yeasts during zinc deprivation. Red: downregulated proteins; Blue: upregulated proteins.

Proteomics showed a rearrangement of metabolic process in order to cope with reduced zinc availability. Enzymes such as hexokinase and phosphofrutokinase-1 were repressed, suggesting a reduction in glycolytic activity in the cell. Proteomic data also revealed that alcohol dehydrogenase (ADH) was suppressed during zinc deprivation, suggesting that the fungus relies on the anaerobic metabolism since ADH is a Zn-dependent enzyme (**Figure 5**). Furthermore, other enzymes related to energy production have also been observed, for example, carnitine-acyl transferase, an important regulator that dictates beta-oxidation. In addition, several enzymes of the citric acid cycle (malate dehydrogenase, succinate dehydrogenase, and isocitrate dehydrogenase) and ATP synthase from oxidative phosphorylation were induced (**Figure 5**). Therefore, these results suggest that the supply of acetyl-CoA for the Krebs cycle in zinc deprivation is not exclusively being provided through the glycolytic pathway but rather by the oxidation of fatty acids. Thus, it is suggested that *H. capsulatum* during zinc deprivation has the ability to modify its energy metabolism to survive the changes imposed by the host. In addition, it has been shown that this fungus has lipid reserves used in adverse conditions, such as during an infectious process. Changes in glycolate biosynthesis (a precursor of pyridoxine biosynthesis), has been revealed by factor analysis. Two enzymes related to pyridoxine production were induced in metal deficiency: pyridoxal 5P kinase and pyridoxal kinase (**Figure 5**). In addition, enzymes related to amino acid degradation such as alanine transaminase, which was induced in zinc deprivation, were also found in this work. This enzyme can help with energy metabolism since it led to the production of pyruvate, that may be directed to TCA cycle.

UDP-N-acetylgalactosamine pyrophosphorylase, an enzyme related to the synthesis of chitin, was induced during zinc deprivation at both time points (24 and 48 h). In addition, we also observed that the glyoxylate cycle was suppressed by zinc deprivation (**Figure 5**). Therefore, it appears that *H. capsulatum* increased the production of structural carbohydrates from glucose that is not consumed by the glycolytic pathway. The consumption of glucose in Zn deprivation is likely related to the remodeling of the wall since glycolysis and fermentation are reduced and beta-oxidation is induced.

### Zinc Deprivation Decreases Fermentation in *H. capsulatum*

**Figure 6:**
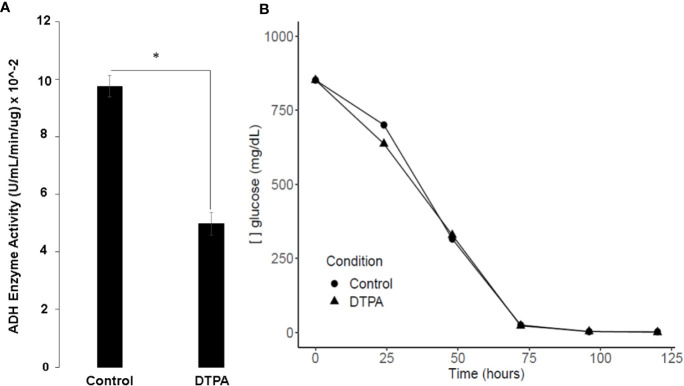
Influence of zinc scarcity glucose metabolism of *H. capsulatum*. **(A)** Enzymatic activity of ADH during zinc deprivation in 24 h. **(B)** Glucose dosage in culture supernatants of cells grown in Zn replete or Zn depleted conditions. All experiments were carried out in biological triplicates. Student t test with *p ≤ 0.05.

The proteomic analysis showed that enzymes such as hexokinase (HCBG_05633), enolase (HCBG_00056), pyruvate carboxylase (HCBG_00107), and alcohol dehydrogenase (HCBG_05406) were repressed in zinc deprivation. Such results indicate that anaerobic metabolism is suppressed at low zinc levels. Thus, to confirm these results, ADH activity was measured in *H. capsulatum* yeasts grown under control conditions and with Zn deficiency (**Figure 6A**). This approach showed that Zn limitation decreases ADH activity, corroborating with proteomic data. Therefore, it is likely that fermentation under conditions of low zinc availability was suppressed in *H. capsulatum*, and probably glucose flow through glycolytic pathway is decreased. In order to confirm that, assays were performed to measure glucose consumption by *H. capsulatum* in zinc deprivation. The glucose consumption did not change in Zn-depleted cells in comparison with control cells (**Figure 6B**), which reinforces the idea that glucose consumed by the fungus is being used for purposes other than energy production.

### Zinc Deprivation Alters the Structural Carbohydrate Distribution in *H. capsulatum*

**Figure 7:**
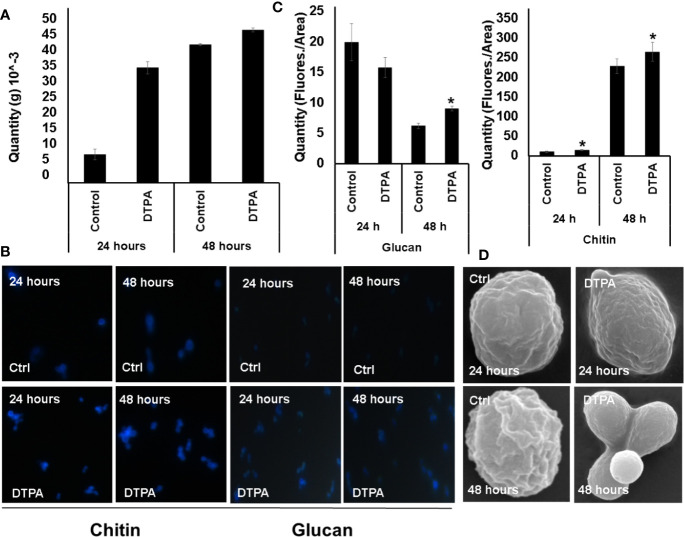
Effect of Zn availability on *H. capsulatum* cell wall carbohydrates. **(A)** Dosage of total carbohydrate in *H. capsulatum* cells during zinc deprivation. **(B)** Fluorescence microscopy *H. capsulatum* grown under control and DTPA conditions. Calcofluor white and aniline blue were used to dosage chitin and glycan, respectively. **(C)** Measurement of glycan and chitin contents using fluorescence intensity. **(D)** Scanning electron microscopy of *H. capsulatum* during zinc deprivation. All experiments were carried out in biological triplicates. The comparisons were made using the Student t test with *p ≤ 0.05.

The decreased levels of glycolytic and fermentative enzymes allied to glucose consumption suggest the fungus may be directing glucose to structural purposes. This hypothesis is supported by an increase in enzymes related to the biosynthesis of wall precursors during Zn deprivation such as UDP-N-acetylglucosamine pyrophosphorylase (HCBG_00326), mannose-6-phosphate isomerase (HCBG_05577), and phosphomannomutase (HCBG_05577). Among them, UDP-N-acetylglucosamine pyrophosphorylase stands out as it is related to chitin biosynthesis. To assess that, *H. capsulatum* carbohydrate content was biochemically measured. Accordingly, an increase in carbohydrate content was observed under 24 h of Zn deprivation. However, after 48 h, there was no significant difference in the amount of carbohydrate present in the cell, suggesting an adaptation of *H. capsulatum* to zinc deprivation over time (**Figure 7A**).

In addition, the increase in the amount of glycan during zinc deprivation may also be related to the production of carbohydrates with structural function. Therefore, it was hypothesized that the glucose consumed by the fungus was being driven to structural carbohydrate biosynthesis. In order to test that hypothesis, the contents of chitin and glycan were measured by fluorescence microscopy. Indeed, Zn limitation induces chitin and glucan accumulation (**Figures 7B, C**). To further confirm those changes, cell wall structure was examined by scanning electron microscopy (**Figure 7D**). The results showed that *H. capsulatum* in the control condition presents a rougher cell wall when compared to zinc deprivation, suggesting that *H. capsulatum* alters cell wall structure and composition during zinc deprivation.

### Zn Limitation and Pyridoxine Synthesis

**Figure 8:**
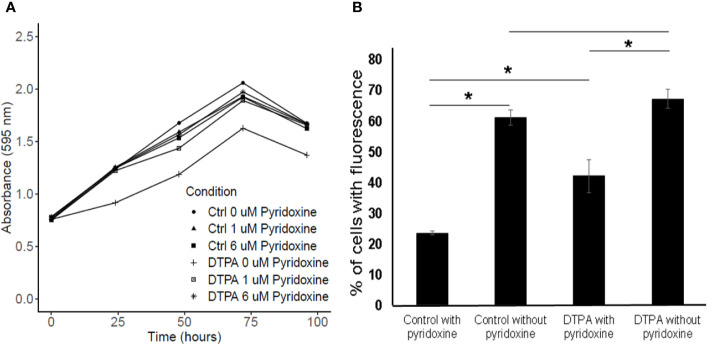
Influence of pyridoxine on *H. capsulatum* growth and oxidative stress level. **(A)** Growth curve of *H. capsulatum* during zinc deprivation at different concentrations of pyridoxine. **(B)** ROS detection of *H. capsulatum* labeled with DCFH-DA. Ctrl, fungal cells grown in zinc; DTPA, fungal cells grown in DTPA. All experiments were carried out in biological triplicates. The comparison was made using the Student t test with *p ≤ 0.05.

Factor analysis results revealed that the glyoxylate reductase (HCBG_03370) was induced during zinc deprivation at 24 h when compared to cells in control condition. However, in 48 h we observed an inversion in the abundancy of the enzyme, in which it is repressed in zinc deficiency. Thus, it appears that the enzyme produced relevant amounts of glycolate during the first 24 h and later, still for unknown reasons, has its quantity reduced. In addition, two enzymes related to vitamin B6 synthesis, pyridoxal 5P synthase and pyridoxal kinase, were identified by proteomic approach. Both were induced after 48 h of zinc deprivation, suggesting an increase in pyridoxine biosynthesis. In order to verify the importance of pyridoxine in *H. capsulatum*, the effect of pyridoxine on fungal growth under Zn limitation was analyzed (**Figure 8A**). Surprisingly, *H. capsulatum* grows similarly when zinc is available regardless of pyridoxine availability. However, the growth in Zn deprivation was affected when no pyridoxine was provided. Therefore, it is suggested that there is an association between zinc homeostasis and vitamin B6 biosynthesis. Furthermore, it is known that pyridoxine is a chemical compound with a high affinity for reactive oxygen species (Matxain et al., 2006). In order to analyze the association of Zn-depletion and ROS stress, ROS labeling assays were performed. The data show that Zn-limitation itself increases the number of labeled cells by twofold. The zinc limitation in absence of pyridoxine potentialize the ROS levels since teh number of labeled cells incresed by threefold. Therefore, it may be concluded that both pyridoxine and zinc are able to influence *H. capsulatum’s* ability to control intracellular ROS levels (**Figure 8B**).

## Discussion

The *in silico* analysis identified eight orthologous genes putatively related to zinc homeostasis in *H. capsulatum*. Two of them are transcription factors (Zap1 and PacC), three belong to the CDF zinc transporter family (Zrc1, Zrc2 and ZitB), and three belong to the ZIP family (Zrt1, Zrt2 and Zrt3). In *A. fumigatus*, zinc homeostasis is regulated by two transcription factors and zinc sensors that control the coordinated action of eight carriers belonging to the ZIP family and eight of the CDF family (Amich and Calera, 2014). In *C. gatti*, zinc carrier proteins of the ZIP family have already been described, such as ZIP1, ZIP2, and ZIP3. These proteins are orthologs of the Zrts/Zrfs found in *H. capsulatum* and *A. fumigatus* (Schneider et al., 2015). As previously mentioned, studies with *H. capsulatum* have shown the presence of a carrier called *Hc*Zrt2 (called here as Zrt1) that behaves in a similar manner to Zrt1 in *S. cerevisiae* (Dade et al., 2016). Thus, we propose the change since most high affinity transporters are named Zrt1, as in *C. albicans* (Crawford et al., 2018)*, C. dubliniensis* (Bottcher et al., 2015)*, B. dermatitidis* (Kujoth et al., 2018), and the already mentioned *S. cerevisiae* (Zhao and Eide, 1996). However, mutant based studies are required to properly characterize the remaining transporters. In addition to zinc uptake mechanisms mediated by carriers of the ZIP and CDF family, a novel zinc uptake protein was first identified in *C. albicans*. Pra1 acts as a “zincophore” similarly to siderophores (Fe carriers) found in bacteria and fungi (Miethke and Marahiel, 2007). This protein holds multiple zinc binding sites in its structure and is able to gather either free or protein associated zinc in the extracellular medium. Studies have shown that Pra1 is normally expressed under low zinc and alkaline conditions, similar to ZrfC in *A. fumigatus* and its orthologs Zrt1 in *C. albicans* where Pra1 has also shown to be associated with both through function, redirecting captured zinc to be transported by ZrfC/Zrt1 (Wilson et al., 2012). However, in *H. capsulatum*, no molecule homologous to Pra1 was found. Thus, from the *in silico* analysis it is suggested that *H. capsulatum* does not have a zinc capture mechanism mediated by zincophore. Finally, while our analyses showed that the genes mentioned are being influenced by zinc to different degrees, mutant studies are required to properly characterize their biological significance in *H. capsulatum*.

During infection, it is known that GM-CSF activated macrophages reduce the availability of free zinc in the *Histoplasma*-containing phagolysosome. This reduction is mediated by the production of metallothioneins that sequester cytoplasmic labile zinc and also pumps out Zn from *H. capsulatum* containing phagolysosome (Vignesh et al., 2013). Corroborating our data showed that fungal survival in macrophages under Zn limitation was decreased when compared to cells under Zn-homeostatic levels. As a successful pathogen, *H. capsulatum* is able to survive and proliferate in such condition. As depicted in this work, it is suggested that this mechanism of zinc capture and management is mediated by carriers of the ZIP and CDF family under the influence of Zap1. Experimentally, *H. capsulatum* captures zinc from the extracellular medium by a high affinity transporter, previously known as HcZrt2 (Dade et al., 2016). In addition, Dade et al. (2016) showed that the fungus in the absence of this gene lose part of its virulence. Therefore, this transporter is one of the evolutionary aspects developed by *H. capsulatum* to capture zinc and increase the chances of survival in hostile environments. As such, it is possible that Zrt1 (HcZrt2), Zrt2, and Zrt3 identified in this work are being induced by low zinc availability, and this induction may be promoted by Zap1.

Currently, it is known that the main mechanism of zinc regulation in fungi is mediated by the transcription factor Zap1. In *S. cerevisiae*, this transcription factor binds to Zinc Responsive Elements (ZREs) found on the promoter regions of genes such as *ZRT1*, *ZRT2*, and *FET3* (Zhao et al., 1998; Eide, 2009). In our gene expression experiments, an increase in Zap1 levels over 24 h was observed, while the high affinity Zn transporter ZRT1, controlled by ZAP1, was induced at 3 h. This uncorrelation of the regulator and its target genes is not a surprise since previous studies have shown that Zap1 protein levels are stable regardless of the transcript amount, being the regulation of zinc transporter mediated not only by Zap1, but also by zinc availability. With it, it is reasonable to assume that as zinc quantity drastically decreases, basal Zap1 levels are enough to quickly induce key transporters before itself. Additionally, expression analysis studies on Zap1 orthologs in previously mentioned fungi showed that the regulator was induced at the 24 h time period (Zhao and Eide, 1997; Schneider et al., 2012; Vicentefranqueira et al., 2018), similar to the results found in the present study. Thus, it is suggested that all these genes are working together to regulate zinc homeostasis, in which Zap1 induces the production of Zrt1, Zrt3, and Zrt2 in the absence of zinc as seen in *S. cerevisiae*, *C. gatti*, and *C. neoformans* (Eide, 2009; Schneider et al., 2012; Wilson et al., 2012; Bottcher et al., 2015). Regarding CDFs, it was observed that the transporters Zrc1 and Zrc2 showed a mild response in the absence of zinc. Thus, more specific studies are required in order to determine the roles of Zrc1 and Zrc2 in zinc homeostasis in *H. capsulatum*.

Proteomic data revealed a change in energy metabolism and biosynthesis of secondary compounds of *H. capsulatum* during zinc deprivation (**Figure 7**). In other fungi, such as *A. fumigatus, C. albicans, C. neoformans, C. gatti, and S. cerevisiae*, bioinformatics studies have shown that 12% of all zinc-binding proteins are involved in metabolic processes of carbohydrates and amino acids (Staats et al., 2013). In this work, changes in enzymes related to glycolysis, fermentation, glyoxylate cycle, amino acid synthesis/degradation, pyridoxine, and glycan biosynthesis were also observed (**Supplementary Tables 02****–****05**). Specifically, in glycolysis, two regulatory enzymes: hexokinase (HCBG_05633) and phosphofrutokinase-1 (HCBG_08430), were repressed, indicating that the glycolytic pathway was oxidizing glucose at a reduced rate. These results corroborate with Medina and Nicholas (1957) who showed a 70% reduction in the activity of *Neurospora crassa* hexokinase in zinc deprivation. Other pathways of energy production, such as beta-oxidation, amino acid degradation, citric acid cycle, and oxidative phosphorylation, were induced by zinc deprivation. Therefore, it is suggested that the energy metabolism remains active, as other sources of energy are being oxidized to compensate the decreased glycolysis activity. Unlike our findings, Parente et al. (2013), utilizing a similar approach, showed that the energy source in *P. brasiliensis* in zinc deprivation occurred through the induction of enzymes in the glycolytic pathway.

Regarding fermentation, our results showed a reduction in the enzymatic activity of alcohol dehydrogenase, indicating a suppression of anaerobic metabolism. These data are consistent with the structural conformation of this protein, since zinc is a fundamental cofactor for the catalytic activity of alcoholic dehydrogenase (Culotta et al., 2006). Additionally, data obtained corroborate with *in silico* results showed by Tristão et al. (2014) in which they analyzed all zinc binding proteins of the *Paracoccidioides* complex, among which were found the enzymes alcohol dehydrogenase and hexokinase. A recent work demonstrated that *H. capsulatum* survival in macrophages is dependent on gluconeogenesis and that glycolysis is dispensable (Shen et al., 2020). Our data demonstrate Zn-depletion decreases glycolysis since the metal structurally composes some glycolytic enzymes. Thus, the non-activation of glycolysis in intraphagosomal milieu may be a phenomenon not only related to low glucose availability but also a result of a Zn poor environment (Vignesh et al., 2013).

**Figure 9:**
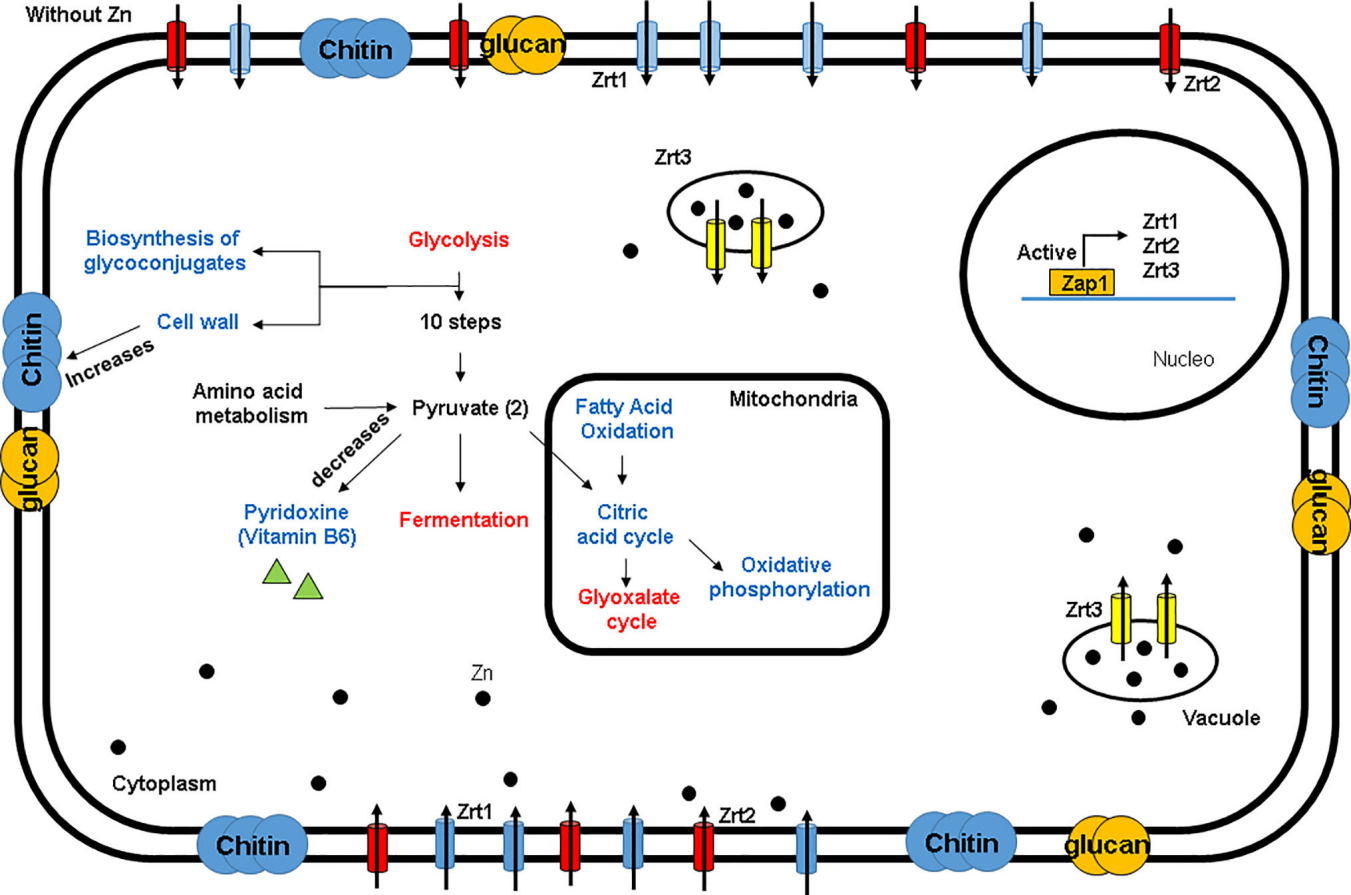
Hypothetical model of *H. capsulatum* response to zinc deprivation.

Experiments on total carbohydrate dosage showed that, when zinc is deprived, the fungus accumulated this compound (**Figure 9**). The main molecules produced were related to structural carbohydrates, since the glycolytic pathway was repressed and enzymes belonging to synthesis of cell wall carbohydrates (Chitin and Glucan) were induced during zinc deprivation. Also, the amount of chitin and glucan in the cell wall of *H. capsulatum* was greater than that of the control. Our results complement those found by Rappleye et al. (2007), which has shown that during infection, *H. capsulatum* processes a large amount of *α*-(1,3)-glucan inside macrophages. High concentrations of glucan inhibit production of cytokines released by the host, such as, tumor necrosis factor alpha (αTNF). Therefore, *H. capsulatum* in the absence of zinc was using glucose for cell wall remodeling since this condition simulate the infectious process. On top of that, scanning microscopy analysis revealed that *H. capsulatum* in normal conditions has a more irregular cell surface when compared to the zinc deprivation condition. The smoother cell surface phenotype may be a direct effect of zinc depletion or a secondary event triggered by metal scarcity. The biological meaning of this cell surface morphology in virulence of *H. capsulatum* will be addressed in further studies. The changes in carbohydrate content differ from those found by de Curcio et al. (2017) in *P. brasiliensis*, in which Zn-limitation promotes a decreasing in chitin content in yeast cells. Such contrasting results may be explained by different pathogenic strategies used by the two fungi. Whereas *H. capsulatum* behaves as an intracellular microorganism, *P. brasiliensis* is not considered a classical intracellular pathogen, being then exposed to different conditions in the host.

Vitamin B6 synthesis reduction is associated with decreased Zn levels in the cell, since the enzyme pyridoxine kinase has a structural site that binds to a Zn–ATP complex (Li et al., 2002). Therefore, it is inferred that under zinc limiting conditions, the enzymatic activity of pyridoxine kinase is altered, resulting in a reduction in pyridoxine synthesis in *H. capsulatum* cells. These results are in agreement with those found by Churchich et al. (1989), in which the enzymatic activity of pyridoxine kinase reduces about 70% during zinc deprivation. This study also showed that pyridoxal kinase can instead be activated by zinc metallothioneins as a rapid response to vitamin B6 biosynthesis in adverse conditions (Karawya and Fonda, 1982). Our data also indicate that oxidative stress is increased in Zn-limitation and is magnified when pyridoxine is not available in the medium. Thus the reduced growth when both Zn and vitamin B6 are reduced is probably due to a cascade effect, in which the lack of zinc reduces the production of vitamin B6 and, as a consequence, increases the amount of reactive oxygen species in *H. capsulatum* cells.

## Conclusions

In this study, eight genes possibly involved in zinc homeostasis were identified in the *in silico* analysis. Proteomic analysis during zinc starvation revealed influence in several metabolic pathways, mainly in the metabolism of glucose, biosynthesis of structural carbohydrates, biosynthesis of pyridoxine and regulation of oxidative stress. Our data also suggest *H. capsulatum* takes glucose for cell wall remodeling. The absence of zinc interferes with pyridoxine biosynthesis, which increases oxidative stress in yeast cells (**Figure 9**). This study sets the groundwork for a deeper understanding of *H. capsulatum* behavior on zinc deprivation, a relevant condition often found during infection. Providing scientists with a new perspective on how *H. capsulatum* is able to thrive on such conditions can help on the establishment of future treatment options.

## Data Availability Statement

The original contributions presented in the study are publicly available. This data can be found here: http://www.ebi.ac.uk/pride, project accession PDX022039.

## Author Contributions

LA, JS, LB, and SB performed the experiments. LA, LS, DM, and MS-B performed data analysis. AB and MS-B designed the research. AB and CS contributed with reagents and/or funds for research. LA, DM, LS, AB, and MS-B contributed to data interpretation and manuscript writing. All authors contributed to the article and approved the submitted version.

## Funding

This work received financial support from the National Council for Scientific and Technological Development (CNPQ, grant number 407666/2016-8), Goiás State Research Support Foundation (FAPEG), Coordination for the Improvement of Higher Education Personnel (CAPES) and the National Institute of Science and Technology of Host-Pathogen Interaction (INCT-IPH).

## Conflict of Interest

The authors declare that the research was conducted in the absence of any commercial or financial relationships that could be construed as a potential conflict of interest.

## Supplementary Material

Supplementary Figure 1 Interaction network of genes related to zinc homeostasis. Zap1 was used as input sequence. Zap1: HCBG_03275; Zrt1: HCBG_07321; Zrt3: HCBG_04549; Zrc1: HCBG_00193; Zrc2: HCBG_00193; ZrfG: HCBG_05775 and ZitB: HCBG_07983. Light blue and purple lines are known interactions from experimental databases. Dark green, red, and dark blue lines are predicted interactions from neighborhood genes, gene fusions, and gene co-occurrence. The light green and black lines are the interactions by co-expression and homology of the protein.

Supplementary Figure 2 DTPA promotes a Zn depleted environment. **(A)** Influence of DTPA on the growth of *H. capsulatum*. Optical density was measured at 595 nm. (**B**) Expression level of *ZRT1* in the control condition, DTPA and DTPA plus Zn (200 µM). (**C)** Expression level of *CTR3* in the control condition, DTPA and DTPA plus Zn (200 µM). All experiments were carried out in biological triplicates. The comparisons were made using the Student t test with *p ≤ 0.05.
