# Supplementary figures and images for: Insights Into Histoplasma capsulatum Behavior on Zinc Deprivation
Source: Front Cell Infect Microbiol. 2020 Nov 30;10:573097. doi: 10.3389/fcimb.2020.573097 (PMC7734293; doi:10.3389/fcimb.2020.573097)

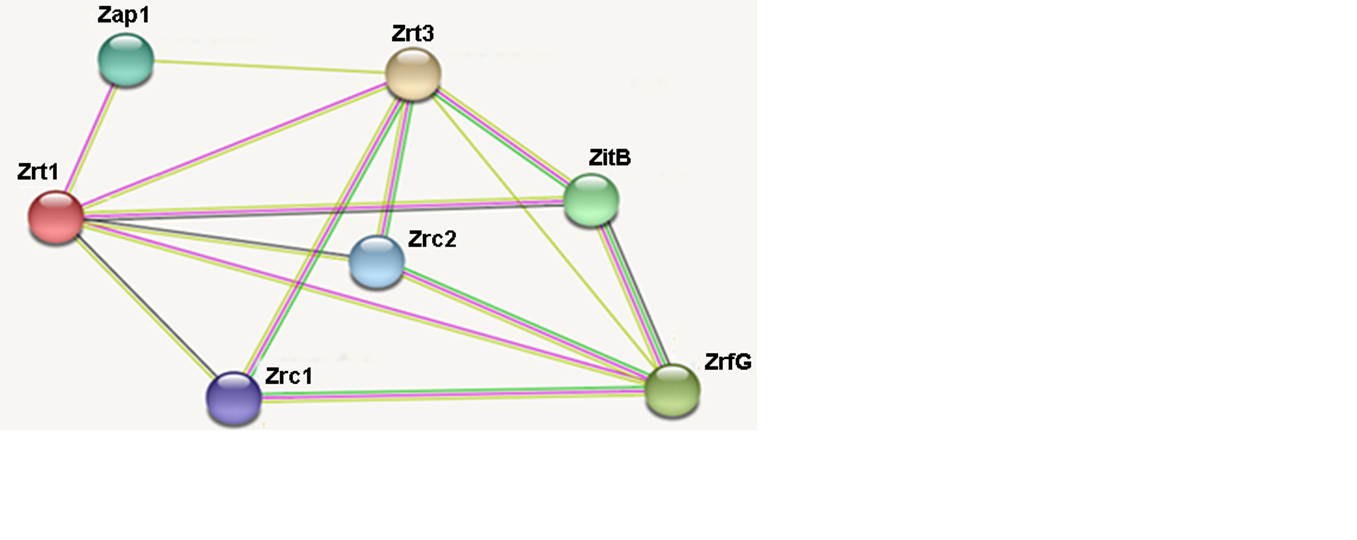

Supplement: Supplementary Figure 1 — Interaction network of genes related to zinc homeostasis. Zap1 was used as input sequence. Zap1: HCBG_03275; Zrt1: HCBG_07321; Zrt3: HCBG_04549; Zrc1: HCBG_00193; Zrc2: HCBG_00193; ZrfG: HCBG_05775 and ZitB: HCBG_07983. Light blue and purple lines are known interactions from experimental databases. Dark green, red, and dark blue lines are predicted interactions from neighborhood genes, gene fusions, and gene co-occurrence. The light green and black lines are the interactions by co-expression and homology of the protein. [file Image_1.tif]

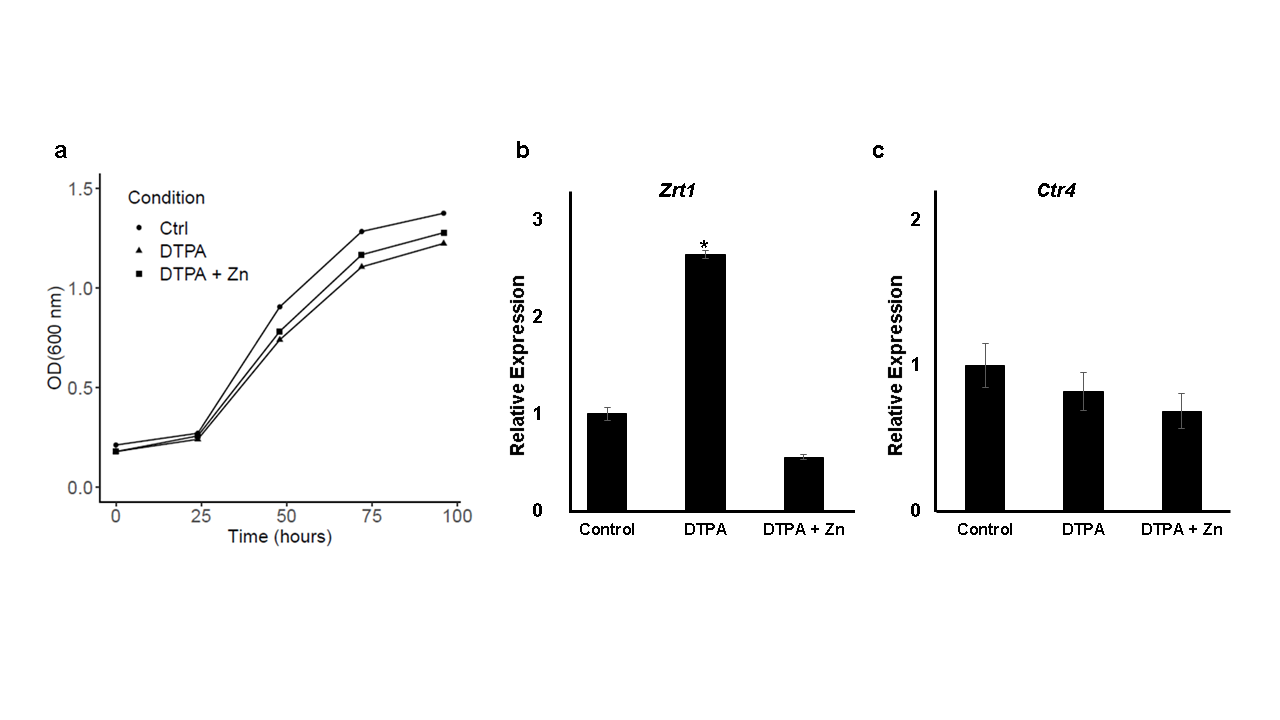

Supplement: Supplementary Figure 2 — DTPA promotes a Zn depleted environment. (A) Influence of DTPA on the growth of H. capsulatum. Optical density was measured at 595 nm. (B) Expression level of ZRT1 in the control condition, DTPA and DTPA plus Zn (200 µM). (C) Expression level of CTR3 in the control condition, DTPA and DTPA plus Zn (200 µM). All experiments were carried out in biological triplicates. The comparisons were made using the Student t test with *p ≤ 0.05. [file Image_2.tif]
