# Supplementary material for: Insights Into Histoplasma capsulatum Behavior on Zinc Deprivation
Source: Front Cell Infect Microbiol. 2020 Nov 30;10:573097. doi: 10.3389/fcimb.2020.573097 (PMC7734293; doi:10.3389/fcimb.2020.573097)
Supplement: Supplementary file 3 [file Table_1.docx]

# ****Supplementary Table 1.**** Oligonucleotides used in this study.

| Accession number | Primer | Sequence (5’-3’) | Tm (°C) |
| --- | --- | --- | --- |
| HCBG_03275 | Zap1 - s | ATCTGCTCGGAAATGCTCTGC | 62 |
|  | Zap1 - as | TCAGCTGGAATTCGGAAGGC | 62 |
| HCBG_07321 | Zrt1 - s | CGGTTCCCAGTCGCCCAG | 62 |
|  | Zrt1 - as | GAATTCGCCAGATGCTCGTG | 62 |
| HCBG_08608 | Zrt2 - s | CAACTGCCTTCATACATCTGC | 62 |
|  | Zrt2 - as | TCCGCCATATTCAACTAGAAAC | 62 |
| HCBG_04549 | Zrt3 - s | GGGTCGAGCATTATCTGTGTG | 62 |
|  | Zrt3 - as | TGTAGAGAGAGCTGAAAAGCAA | 62 |
| HCBG_00193 | Zrc1 - s | GCTTCGTCTCATTGGAAAGGG | 62 |
|  | Zrc1 - as | CTCGGGCGCCATGGTTGTG | 62 |
| HCBG_02465 | Zrc2 - s | CTCCTTGAACTAGTCGTTGGTT | 62 |
|  | Zrc2 - as | GCCCGTTGCCACCCATAGG | 62 |
| HCBG_06240 | Ctr3 - a | GGACTGTATTGAATGCCTGTTT | 62 |
|  | Ctr3 - as | ATCGTCCGGTATAGAAGTTGC | 62 |
| HCBG_08711 | Actin - s | GTCCTCGCCATCATGGTATTA | 62 |
|  | Actin - as | CTCAGGAGCGACACGGAGT | 62 |
